# Imaging and Clinicopathological Features of Squamous Cell Metaplastic Breast Carcinoma: A 22-Case Retrospective Study

**DOI:** 10.3390/jcm15083157

**Published:** 2026-04-21

**Authors:** Feng Pan, Lei Liu, Dingbao Chen, Yuan Peng

**Affiliations:** 1 Department of Radiology, Peking University People’s Hospital, Beijing 100044, China; panfeng@hsc.pku.edu.cn (F.P.);; 2 Department of Pathology, Peking University People’s Hospital, Beijing 100044, China; 3 Breast Center, Peking University People’s Hospital, Beijing 100044, China

**Keywords:** squamous cell metaplastic breast carcinoma, triple-negative breast cancer, magnetic resonance imaging, prognosis, recurrence

## Abstract

**Background/Objectives**: Squamous cell metaplastic breast carcinoma (SCMBC) is a rare and aggressive breast cancer subtype with limited imaging and prognostic data. This study aimed to characterize the multimodal imaging features, clinicopathological profiles, and prognostic outcomes of SCMBC in a single-center cohort. **Methods**: We retrospectively analyzed 22 patients with histopathologically confirmed SCMBC treated between January 2012 and May 2025. Clinicopathological profiles, multimodal imaging features, and prognostic outcomes were collected and evaluated. **Results**: All patients were female (median age 64.5 years; range, 34–82). The majority (59.1%) presented with clinical stage II disease. Axillary lymph node metastasis was present in eight (36.4%) patients at diagnosis, with one case of distant lung metastasis. The mean tumor diameter was 4.24 cm (range, 2.1–11.8). MRI findings (*n* = 13) included heterogeneous internal structure, a low mean apparent diffusion coefficient (ADC) value of 0.98 × 10^−3^ mm^2^/s, and frequent necrosis (92.3%). Pathologically, 54.5% of tumors were high-grade, and 81.8% exhibited a triple-negative phenotype. After a median follow-up of 34 months, the 5-year overall survival (OS) rate was 64.3%, while the 3-year disease-free survival (DFS) rate was 42.1%. The recurrence/metastasis rate was 22.7% (5/22), and all five deaths occurred in this subgroup. **Conclusions**: SCMBC is characterized by suggestive multimodal imaging features, a predominant triple-negative phenotype, and a high risk of early recurrence. Given the exploratory nature of this single-center study with a limited sample size, these findings require validation in larger, prospective cohorts. Early radiological identification and aggressive personalized treatment strategies may improve outcomes for patients with this aggressive disease.

## 1. Introduction

Metaplastic breast carcinoma (MBC) is a rare and histologically heterogeneous group of malignancies, accounting for approximately 0.2% to 5.0% of all invasive breast cancers [1,2]. Among its various subtypes, squamous cell metaplastic breast carcinoma (SCMBC) is one of the most common, defined by the presence of unequivocal squamous differentiation [3,4].

Clinically and immunophenotypically, SCMBC often overlaps with triple-negative breast cancer (TNBC); however, it is generally characterized by more aggressive biological behavior and a less favorable prognosis [5]. Despite its clinical significance, the current literature on SCMBC remains substantially limited. Most available studies are confined to case reports or small case series, leading to a fragmented understanding of its radiopathological features [6,7]. Recent larger studies have begun to characterize metaplastic breast cancer more comprehensively. For instance, Yao et al. reported multimodal imaging features in a cohort of metaplastic breast cancer patients, and Zhang et al. described clinicopathological characteristics in a pure squamous cell cohort [8,9]. However, a detailed integration of multimodal imaging—specifically quantitative MRI parameters such as apparent diffusion coefficient (ADC) values—with comprehensive pathological and survival data remains lacking.

Current diagnostic methods for SCMBC face significant challenges. Preoperative imaging often fails to distinguish SCMBC from benign conditions such as abscesses or from other triple-negative breast cancers, leading to diagnostic delays or inappropriate surgical planning [10]. In breast oncology, ADC values correlate with tumor aggressiveness, with more aggressive lesions typically showing lower ADC values [11]. In IDC, typical ADC values range from 1.0 to 1.3 × 10^−3^ mm^2^/s, reflecting moderate cellularity. In contrast, preliminary evidence suggests that SCMBC may exhibit lower ADC values due to its high cellular density and squamous differentiation, yet systematic characterization is lacking. Establishing a reliable imaging signature for SCMBC could improve preoperative diagnostic accuracy and guide more appropriate treatment strategies.

Emerging targeted therapy strategies for SCMBC remain an area of active investigation. Given the aggressive nature and chemoresistance of this subtype, there is growing interest in therapies directed at tumor biology. The high prevalence of EGFR expression (observed in 59.1% of our cohort) suggests potential vulnerability to EGFR inhibitors such as erlotinib or gefitinib, which have shown activity in other squamous cell carcinomas. Additionally, antibody–drug conjugates (e.g., sacituzumab govitecan, which targets Trop-2) and immune checkpoint inhibitors are being explored in triple-negative breast cancer and may hold promise for SCMBC [2]. Ongoing clinical trials are evaluating these agents in metaplastic breast cancer, offering potential therapeutic avenues for this challenging disease.

To address these gaps, we conducted a retrospective analysis of 22 patients with pathologically confirmed SCMBC at a single institution in Beijing. While our findings reflect the clinical practices and patient demographics of a Chinese population, they provide a foundational characterization that can be compared and validated in future multicenter studies. We hypothesized that SCMBC would exhibit distinct MRI features, including lower apparent diffusion coefficient (ADC) values and a higher prevalence of necrosis compared to non-metaplastic triple-negative breast cancer, and that these imaging features would correlate with aggressive pathological phenotypes and poor prognosis. This study aims to (1) comprehensively delineate the clinicopathological and multimodal imaging characteristics of this tumor, with emphasis on its MRI features including diffusion-weighted imaging; (2) evaluate prognostic factors and survival outcomes; and (3) provide consolidated evidence to aid in early diagnosis, risk stratification, and the formulation of personalized treatment strategies for this challenging disease.

## 2. Materials and Methods

### 2.1. Study Population and Design

This retrospective, single-center cohort study was conducted in accordance with the Declaration of Helsinki. The requirement for informed consent was waived by the Institutional Review Board of Peking University People’s Hospital (Approval No. 2026PHB157-001) due to the retrospective and anonymized nature of the data analysis. We identified patients with pathologically confirmed SCMBC who were treated at our institution between January 2012 and May 2025. The inclusion criteria were as follows: (1) histopathological confirmation of SCMBC, and (2) availability of pretreatment breast MRI, ultrasound, or mammography with evaluable imaging data. Clinical data collected included patient age, clinical tumor stage, neoadjuvant chemotherapy status, surgical procedure, overall survival (OS), and disease-free survival (DFS).

### 2.2. Imaging Acquisition

#### 2.2.1. Breast MRI

All MRI examinations were performed using a 3.0 T GE Discovery 750 scanner (GE Healthcare, Chicago, IL, USA) with a dedicated breast coil. The imaging protocol included axial T1-weighted and fat-saturated T2-weighted sequences, diffusion-weighted imaging (DWI; b-values: 0 and 800 s/mm^2^), and dynamic contrast-enhanced (DCE) MRI. DCE-MRI was acquired with a slice thickness of 1 mm and no gap following the intravenous administration of gadopentetate dimeglumine at a dose of 0.2 mL/kg and an injection rate of 2 mL/s.

#### 2.2.2. Breast Ultrasound

Examinations were conducted using GE Discovery ultrasound systems (GE Healthcare, Chicago, IL, USA) equipped with 7–10 MHz linear array transducers. Standardized bilateral breast and axillary evaluations were performed with the patient in the supine position.

#### 2.2.3. Mammography

Digital mammography was performed using a GE Senographe 2000 D system (GE Healthcare, Chicago, IL, USA). Standard craniocaudal (CC) and mediolateral oblique (MLO) views were obtained for all patients.

### 2.3. Image Analysis

All imaging studies were independently reviewed by two breast radiologists, each with over 10 years of experience, who were blinded to the clinical outcomes and the final pathological diagnosis. The MRI evaluation encompassed tumor size, morphology, margin characteristics, internal signal intensity (T1-, T2-weighted, and DWI), the presence of necrosis, enhancement kinetics, and quantitative apparent diffusion coefficient (ADC) values, which were measured by placing three circular regions of interest (ROIs) of approximately 30–50 mm^2^ on the most restricted diffusion areas of the tumor, carefully avoiding necrotic, cystic, or hemorrhagic regions. ADC maps were reconstructed using a mono-exponential model with b-values of 0 and 800 s/mm^2^. The mean ADC value was calculated from these three measurements. Ultrasound assessment included size, echogenicity, margins, posterior acoustic features, vascularity (graded using the Adler classification), and axillary nodal status. Mammographic evaluation focused on mass density, the presence of calcifications, and architectural distortions. Inter-observer agreement was assessed using Cohen’s kappa coefficient for key imaging features (e.g., presence of necrosis, time–intensity curve pattern), yielding a kappa value of 0.82, indicating excellent agreement. For any discrepant interpretations, a consensus was reached through discussion with a third senior radiologist.

### 2.4. Pathological Evaluation

The diagnosis of SCMBC was confirmed on surgical resection specimens. Tissue samples were fixed in 10% neutral buffered formalin, embedded in paraffin, and sectioned for routine hematoxylin and eosin (H&E) staining. Unequivocal squamous differentiation was defined by the presence of at least one of the following criteria: intercellular bridges, keratin pearl formation, and/or immunohistochemical expression of squamous markers (CK5/6, p63). Immunohistochemical (IHC) staining was performed using the EnVision™ FLEX system. Estrogen receptor (ER) and progesterone receptor (PR) positivity were defined as nuclear staining in ≥1% of tumor cells. HER2 status was interpreted according to the Chinese Anti-Cancer Association guidelines: scores of 0 or 1+ were considered negative; a score of 2+ was considered equivocal and required mandatory fluorescence in situ hybridization (FISH) for confirmation; and a score of 3+ was defined as positive.

### 2.5. Statistical Analysis

Statistical analyses were performed using SPSS software (version 26.0). Survival outcomes, including OS and DFS, were estimated using the Kaplan–Meier method and compared with the log-rank test. Prognostic factors were assessed using multivariable Cox proportional hazards regression models. Continuous variables are presented as median (range), and categorical variables are presented as frequencies (%). Results from the regression analyses are reported as hazard ratios (HR) with 95% confidence intervals (CI). A two-tailed *p*-value of <0.05 was considered statistically significant.

## 3. Results

### 3.1. Clinical Characteristics and Survival Outcomes

A total of 22 female patients with a median age of 64.5 years (range, 34–82) were included in this study. The clinical stage distribution is detailed in Table 1: 12 patients (54.5%) were stage IIA (T2N0M0), 6 (27.3%) were stage IIB (4 T2N1M0, 2 T3N0M0), 3 (13.6%) were stage IIIA (T2N2M0, T4N1M0), and 1 (4.5%) was stage IV (T4N1M1) with pulmonary metastasis at diagnosis.

Surgical interventions included modified radical mastectomy (*n* = 13, 59.1%), total mastectomy with sentinel lymph node biopsy (*n* = 7, 31.8%), and breast-conserving surgery with axillary dissection (*n* = 2, 9.1%). Ten patients (45.5%) received neoadjuvant chemotherapy, and nine (40.9%) underwent postoperative radiotherapy. Axillary lymph node metastases were present in eight patients (36.4%) at initial diagnosis.

**Figure 1:**
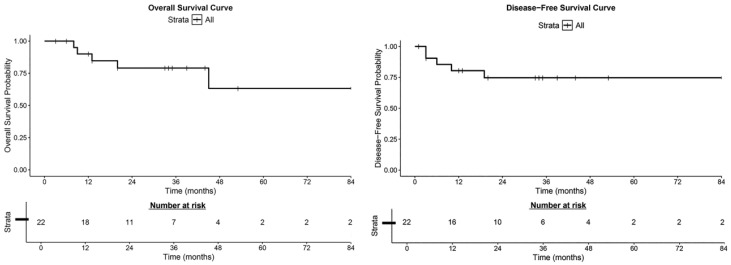
Kaplan–Meier analysis of overall survival (OS) and disease-free survival (DFS). The Kaplan–Meier method was used to assess overall survival (OS) and disease-free survival (DFS) in the study cohort. Follow-up time is presented in months. Survival curves (solid lines) indicate cumulative survival rates, and vertical ticks (│) represent censored data. The risk table shows the number of remaining cases at each time point. Statistical analysis revealed a median OS of 53 months (95% CI: 34–NA) and a median DFS of 34 months (95% CI: 19–53).

After a median follow-up of 34 months (range, 3–87), recurrence events were observed in five patients (22.7%), including one with local chest wall recurrence, three with distant metastases (to lung, liver, or bone), and one with combined local and distant recurrence. All five deaths occurred in this subgroup. The 5-year overall survival (OS) rate was 64.3%, and the 3-year disease-free survival (DFS) rate was 42.1% (Figure 1). Among the five patients with recurrence, three received salvage therapies (including second-line chemotherapy and/or radiotherapy), which contributed to prolonged survival despite early disease recurrence. Sensitivity analysis excluding the single Stage IV patient showed minimal changes in survival estimates (3-year DFS: 42.9%; 5-year OS: 66.7%), indicating that this outlier did not substantially skew the main findings.

In the multivariable Cox regression analysis, which was adjusted for age, clinical stage, histologic grade, molecular subtype, Ki-67 index, neoadjuvant treatment response, and recurrence/metastasis status, the proportional hazards assumption was met (Schoenfeld residuals global test *p* = 0.34). The model demonstrated good discriminative ability (Harrell’s C-index = 0.82). Recurrence or metastasis was identified as the only independent predictor of mortality (adjusted Hazard Ratio [HR] = 7.15, 95% Confidence Interval [CI]: 1.01–50.6; *p* = 0.049). The wide confidence interval reflects the uncertainty inherent in the small sample size; therefore, this finding, while statistically significant, should be interpreted with caution. A trend towards increased mortality risk was observed in patients with progressive disease (PD) after neoadjuvant chemotherapy (HR = 4.95, 95% CI: 0.77–31.8; *p* = 0.091). Given the limited number of death events (*n* = 5), the multivariable model is at risk of overfitting; therefore, these findings should be interpreted as exploratory and require validation in larger cohorts (Table 2).

### 3.2. Imaging Findings

**Figure 2:**
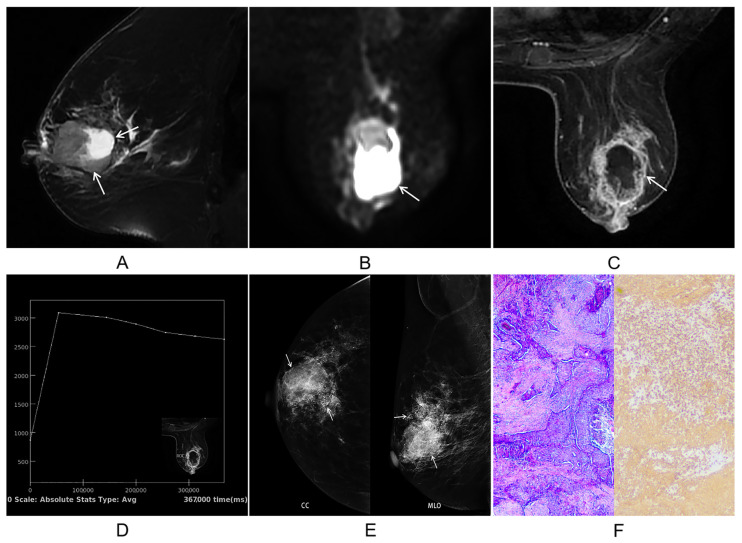
A 65-year-old female with right breast metaplastic squamous cell carcinoma. (**A**) Sagittal T2-weighted image demonstrates a mass with heterogeneous iso- to hyperintense signal and internal necrotic areas (arrows). (**B**) Diffusion-weighted imaging reveals marked hyperintensity (arrows), indicating restricted diffusion. (**C**) Dynamic contrast-enhanced MRI shows rim enhancement of the tumor (arrows). (**D**) Time–signal intensity curve demonstrates washout kinetics. (**E**) Mammography (CC (craniocaudal) and MLO (mediolateral oblique) views) views) reveals a mass with associated clustered microcalcifications (arrows). (**F**) Histopathological examination (H&E and CK5 staining, ×100) shows tumor cells arranged in nested and cord-like patterns, with strong CK5 immunoreactivity.

The mean tumor diameter on imaging was 4.24 cm (range, 2.1–11.8 cm). Among the 13 patients who underwent MRI, the lesions typically demonstrated heterogeneous signal intensity, appearing isointense to hypointense on T1-weighted imaging and heterogeneously hyperintense on T2-weighted imaging in the majority of cases (12/13, 92.3%) (Figure 2A). Diffusion-weighted imaging (DWI) showed restricted diffusion, appearing hyperintense on high b-value images (Figure 2B), with a corresponding low mean apparent diffusion coefficient (ADC) value of 0.98 × 10^−3^ mm^2^/s (range, 0.768–1.298 × 10^−3^ mm^2^/s). Necrosis was a common feature, observed in 12 of 13 lesions (92.3%), manifesting as extensive intratumoral necrosis (>50% of the tumor area, *n* = 8) (Figure 2C), focal necrosis (*n* = 1), or peripheral cystic degeneration (*n* = 3). The time–intensity curves (TIC) from dynamic contrast-enhanced MRI displayed washout (6/13, 46.2%) (Figure 2D) or plateau (7/13, 53.8%) patterns.

Ultrasound examination (*n* = 19) revealed solid masses that were predominantly hypoechoic (13/19, 68.4%) or of mixed echogenicity (6/19, 31.6%). Most lesions demonstrated irregular shape (15/19, 78.9%) and non-parallel orientation (14/19, 73.7%), with spiculated margins observed in 8 cases (42.1%). Posterior acoustic features included enhancement (9/19, 47.4%), no significant change (6/19, 31.6%), or attenuation (4/19, 21.1%). Vascularity, as assessed by means of Doppler, was moderate to marked (Adler grade ≥ 3) in 13 cases (68.4%).

Mammography (*n* = 18) identified malignant calcifications (clustered or amorphous) in 7 cases (38.9%), which were predominantly clustered (5/7, 71.4%) with fine pleomorphic or amorphous morphology (Figure 2E). Associated architectural distortion was observed in 6 cases (33.3%). Imaging of distant metastatic sites revealed multiple pulmonary nodules, hypovascular hepatic lesions, and osteolytic bone destruction.

### 3.3. Pathological and Immunohistochemical Findings

Histopathological examination revealed that the tumors were composed of neoplastic cells arranged in nests or cord-like structures (Figure 2F), with keratin pearl formation observed in moderately to well-differentiated areas. However, focal regions frequently exhibited marked cellular atypia and a high mitotic rate. Accordingly, 12 tumors (54.5%) were classified as histological grade III and 10 (45.5%) as grade II. Tumor necrosis (both geographic and cystic patterns) accompanied by lymphocytic and neutrophilic infiltration was a frequent finding.

Lymphovascular invasion was identified in eight cases (36.4%), and perineural invasion was also noted. Lymph node metastases were confirmed in eight patients, with one case showing extensive nodal involvement (19 out of 24 nodes positive). Immunohistochemically, the majority of tumors (18/22, 81.8%) exhibited a triple-negative phenotype (ER-, PR-, HER2-). The characteristic immunoprofile included consistent positivity for CK5/6 (22/22, 100%) (Figure 2F), expression of p63 in 17 cases (77.3%), and EGFR positivity in 13 cases (59.1%). The Ki-67 proliferation index varied widely from 10% to 80%, with a median of 35% and a mean of 42.3%; 12 cases (54.5%) had a Ki-67 index exceeding 30%.

## 4. Discussion

Squamous cell metaplastic breast carcinoma represents a formidable diagnostic and therapeutic challenge due to its rarity, pathological complexity, and aggressive clinical behavior. Our study, comprising one of the larger single-center cohorts of SCMBC, provides comprehensive insights into its clinical trajectory, multimodal imaging hallmarks, and distinct pathological profile.

Consistent with the prior literature, our cohort consisted exclusively of female patients with a median age of 64.5 years, an age profile similar to other metaplastic carcinomas but notably older than that for common invasive ductal carcinoma [12]. A striking finding was the large tumor size at presentation (mean diameter > 4 cm), with nearly half of the patients (45.5%) presenting at clinical stage IIB or higher [13]. This aligns with reports suggesting that SCMBC is often diagnosed at an advanced T-stage. The high propensity for early systemic dissemination was evidenced by 36.4% of patients having axillary nodal metastases at diagnosis and one patient presenting with synchronous distant metastases. Despite aggressive local therapy, with the majority undergoing mastectomy, we observed a discouraging 3-year DFS of 42.1%, starkly highlighting the tumor’s virulent nature [14,15].

Our multivariable analysis identified postoperative recurrence or metastasis as the sole independent predictor of mortality, with a seven-fold increased risk of death. This underscores the pivotal role of effective systemic control in determining long-term survival. While some studies have identified lymph node metastasis as a key prognostic factor [16], our data did not confirm this. Several biological factors may contribute to this discrepancy. First, the overriding prognostic impact of distant relapse may overshadow nodal status in SCMBC, as hematogenous spread may represent a more dominant pathway for this aggressive subtype. Second, SCMBC, like other basal-like breast cancers, may have a higher propensity for hematogenous rather than lymphatic dissemination. Third, our limited sample size may have underpowered the detection of a true association. These observations suggest that in SCMBC, systemic control may be more critical than regional nodal management. A strong, albeit borderline significant, trend was also observed for progressive disease after neoadjuvant chemotherapy, suggesting that chemoresistance is a major clinical hurdle [17]. Notably, 50% of patients receiving neoadjuvant chemotherapy achieved only stable disease, which, given the aggressive nature of SCMBC, may further reflect chemoresistance—a finding consistent with previous reports on metaplastic breast cancer [18].

The aggressive biology of SCMBC is reflected in its characteristic imaging features. On MRI, the tumors consistently showed heterogeneous architecture, a reflection of intratumoral necrosis and hypercellular components [19]. The low mean ADC value (0.98 × 10^−3^ mm^2^/s) is a critical finding, indicating high cellularity and dense structure, and serves as a key differentiator from benign mimics like abscesses, where necrotic components typically show facilitated diffusion [20]. The high prevalence of necrosis (92.3% on MRI), often extensive, is a radiological signature of rapid tumor growth outstripping its blood supply [10]. The malignant vascular phenotype was further confirmed by the predominantly washout and plateau kinetic curves on DCE-MRI [19]. Ultrasound and mammography remain important first-line tools for breast cancer evaluation. In our cohort, ultrasound typically revealed hypoechoic masses with variable posterior acoustic features, and mammography identified malignant calcifications in 38.9% of cases. However, MRI offered distinct advantages in characterizing SCMBC, particularly in assessing internal architecture, necrosis patterns, and providing quantitative ADC values that aid in preoperative differentiation from benign mimics such as abscesses. While ultrasound and mammography provide complementary information, our experience confirms that MRI offers superior soft-tissue characterization and is a valuable adjunct for staging and surgical planning [21]. This is particularly important given that MRI was performed in only 59% of our patients; in settings where MRI is not readily available, ultrasound and mammography remain essential for initial assessment and follow-up.

Comparison with other breast cancer subtypes reveals distinct features of SCMBC. Compared with invasive ductal carcinoma (IDC) of no special type, SCMBC in our cohort presented at an older age (median 64.5) and with larger tumors (mean 4.24 cm). The predominantly triple-negative phenotype (81.8%) aligns with the aggressive behavior of basal-like breast cancers, but the 3-year DFS of 42.1% is notably worse than that reported for non-metaplastic triple-negative breast cancer (approximately 70–80%) [22]. Compared with other metaplastic carcinoma variants, the squamous subtype showed a higher prevalence of necrosis on MRI (92.3%) and a higher rate of EGFR expression (59.1%), suggesting potential biological differences that may influence therapeutic strategies.

The correlations between pathological and imaging findings in SCMBC are deeply rooted in the tumor’s underlying biology, a concept increasingly recognized in breast cancer diagnostics [22]. In our study, the low mean ADC value (0.98 × 10^−3^ mm^2^/s) corresponds histopathologically to high cellular density and densely packed tumor cells with scant intervening stroma, reflecting the high-grade nature of SCMBC. Similarly, the extensive necrosis observed on MRI (92.3% of lesions) correlates with the rapid proliferation rate (Ki-67 index > 30% in 54.5% of cases) and aggressive growth outstripping vascular supply. The predominantly washout and plateau kinetic curves on DCE-MRI are consistent with the angiogenic phenotype characteristic of basal-like breast cancers, driven by vascular endothelial growth factor (VEGF) overexpression and aberrant microvessel formation. These imaging-biology correlations are clinically relevant, as they enable preoperative identification of aggressive tumor behavior and may guide treatment intensification. For instance, the presence of low ADC values and extensive necrosis on MRI could serve as imaging biomarkers to identify patients who may benefit from more aggressive systemic therapy or enrollment in clinical trials of novel agents targeting angiogenesis or proliferation pathways.

Pathologically, the diagnosis is anchored on the demonstration of squamous differentiation. The predominant triple-negative immunophenotype (81.8%) and high Ki-67 index (median 35%) in our cohort are hallmarks of its aggressive biology. The consistent expression of basal markers (CK5/6 100%, p63 77.3%) not only confirms the diagnosis but also places SCMBC within the spectrum of basal-like breast cancers. The frequent EGFR positivity (59.1%) observed in our patients is a noteworthy finding, pointing to a potential therapeutic vulnerability that warrants exploration in clinical trials [2]. Based on our findings, we propose several specific strategies for aggressive personalized management. First, for patients with high-risk features (large tumors, high Ki-67 index, or incomplete response to neoadjuvant therapy), consideration should be given to adjuvant capecitabine, extrapolated from the CREATE-X trial experience in residual triple-negative breast cancer. Second, given the high EGFR expression rate (59.1%) in our cohort, evaluation of EGFR status should be considered to guide potential enrollment in clinical trials of EGFR inhibitors (e.g., erlotinib, gefitinib) or antibody–drug conjugates targeting EGFR. Third, post-treatment surveillance should include regular imaging, preferably MRI, every 6 to 12 months for at least 3 years, given the high early recurrence rate (3-year DFS of 42.1%) observed in this study.

The clinical relevance of SCMBC tumor biology extends beyond potential therapeutic targets to encompass prognosis, survival, and metastatic patterns. The predominantly triple-negative phenotype (81.8% in our cohort) inherently confers a more aggressive clinical course compared to hormone receptor-positive subtypes, with higher rates of distant recurrence and shorter overall survival [23]. The high Ki-67 proliferation index (median 35%) observed in our patients further corroborates the rapid growth kinetics of SCMBC, which aligns with the early recurrence events (3-year DFS of 42.1%) documented in this study. Notably, the distinct metastatic pattern of SCMBC—predominantly hematogenous spread to lung, liver, and bone, as observed in our cohort—may be explained by its basal-like molecular profile, which is associated with enhanced epithelial-to-mesenchymal transition (EMT) and cancer stem cell features. These biological characteristics not only inform prognosis but also guide clinical decision-making, suggesting that systemic control should be prioritized over regional management in this aggressive subtype. Regarding marker expression, while CK5/6 and p63 positivity confirmed squamous differentiation, EGFR positivity (59.1%) was associated with a trend toward poorer prognosis, though this did not reach statistical significance. Larger studies are needed to confirm whether EGFR expression serves as an independent prognostic factor or predictive biomarker for targeted therapy in SCMBC.

As a single-center study conducted at a tertiary hospital in Beijing, China, our findings reflect the clinical practices, treatment protocols, and patient demographics specific to this region. The management of breast cancer in China may differ from that in other countries in terms of treatment guidelines, availability of targeted therapies, and patient genetic backgrounds. Therefore, the generalizability of our findings to other populations may be limited. Multicenter, international studies with larger cohorts are needed to validate our observations and determine whether the imaging and pathological features of SCMBC described here are consistent across diverse populations.

Future research directions should prioritize several key areas. First, validation of our findings in larger, multi-institutional prospective cohorts is essential to confirm the prognostic value of MRI features (low ADC values, necrosis) and the independence of recurrence/metastasis as a predictor of mortality. Second, investigation of the molecular mechanisms underlying chemoresistance in SCMBC is needed to identify potential therapeutic targets; the high rate of stable disease (50%) in our neoadjuvant cohort highlights this urgent need. Third, targeted therapies warrant exploration, particularly EGFR inhibitors given the 59.1% expression rate in our cohort, as well as antibody–drug conjugates (e.g., sacituzumab govitecan targeting Trop-2) and immune checkpoint inhibitors that have shown promise in triple-negative breast cancer. Fourth, prospective studies incorporating molecular profiling (e.g., genomic, transcriptomic, and proteomic analyses) are needed to better understand the biological drivers of SCMBC and to identify predictive biomarkers for treatment selection. Ultimately, these efforts aim to improve outcomes for patients with this challenging disease.

## 5. Conclusions

In conclusion, SCMBC is a highly aggressive variant of triple-negative breast cancer that predominantly affects older women and is characterized by rapid progression and a high risk of early recurrence. Despite the limitations inherent in its retrospective, single-center design and small sample size, this study provides suggestive radiological and pathological clues that may aid in recognition of this rare entity. Key radiological clues include large tumor size with heterogeneous internal architecture, restricted diffusion (low ADC values), and extensive necrosis on MRI. Pathologically, it is defined by squamous differentiation, a basal-like immunoprofile, and a high proliferation index. The strong association between recurrence/metastasis and mortality underscores the critical importance of preventing recurrence through effective initial therapy and early detection through vigilant surveillance. Based on our findings, we propose specific clinical management strategies: consideration of adjuvant capecitabine for high-risk patients, evaluation of EGFR status to guide potential targeted therapy trials, and regular imaging surveillance (preferably MRI) every 6 to 12 months for at least 3 years. Future efforts should focus on validating these prognostic factors in larger, multi-institutional cohorts to confirm the preliminary findings reported herein.

## 6. Study Limitations

This study has several limitations that should be acknowledged. First, as a single-center retrospective analysis with a relatively small sample size (*n* = 22), there is an inherent risk of selection bias, and the statistical power for some subgroup analyses is limited. The small number of death events (*n* = 5) restricts the robustness of multivariable analyses; therefore, our findings should be interpreted as exploratory. Second, the study period spans 13 years (2012–2025), during which advancements in imaging technology and pathological testing standards may have introduced variability in data acquisition and interpretation. Third, not all patients underwent all three imaging modalities (e.g., only 13 of 22 patients had MRI), leading to inconsistent data density across modalities and limiting the strength of multimodal comparisons. Fourth, the absence of a comparative control group (e.g., non-metaplastic triple-negative breast cancer) precludes definitive identification of features truly unique to SCMBC. Finally, as a single-institution study from China, the generalizability of our findings to other populations may be limited. Future multicenter prospective studies with larger cohorts are needed to validate our findings.

## Figures and Tables

**Table 1:** Clinicopathological characteristics of patients with squamous cell metaplastic breast carcinoma (*n* = 22).

Variable	Value/*n* (%) or Median (Range)
Demographics	
Age, years	64.5 (34–82)
Sex, female	22 (100)
Clinical stage	
I/II	18 (81.8)
III/IV	4 (18.2)
Treatment	
Neoadjuvant chemotherapy	10 (45.5)
Response: PR	3 (30.0)
Response: SD	5 (50.0)
Response: PD	2 (20.0)
Surgery	
Mastectomy + ALND	13 (59.1)
Mastectomy + SLNB	7 (31.8)
BCS + ALND	2 (9.1)
Postoperative RT	9 (40.9)
Pathology	
Histologic grade	
Grade 2	8 (36.4)
Grade 3	14 (63.6)
Molecular subtype	
Triple-negative	18 (81.8)
HER2-positive	1 (4.5)
HR-positive	3 (13.6)
Ki-67 index	
<30%	10 (45.5)
≥30%	12 (54.5)

Abbreviations: ALND, axillary lymph node dissection; SLNB, sentinel lymph node biopsy; BCS, breast-conserving surgery; RT, radiotherapy; PR, partial response; SD, stable disease; PD, progressive disease; HR, hormone receptor.

**Table 2:** Multivariable Cox regression analysis of factors associated with overall survival.

Variable	Comparison	HR (95% CI)	*p*-Value
Age	Per 1-year increase	1.02 (0.93–1.11)	0.721
Histologic grade	Grade 3 vs. Grade 2	2.89 (0.42–19.9)	0.283
Molecular subtype	TNBC vs. non-TNBC	1.88 (0.18–19.2)	0.598
Ki-67 index	≥30% vs. <30%	1.47 (0.22–9.82)	0.689
Clinical stage	III–IV vs. I–II	3.62 (0.54–24.3)	0.186
Neoadjuvant response	PD vs. PR/SD	4.95 (0.77–31.8)	0.091
Recurrence/Metastasis	Yes vs. No	7.15 (1.01–50.6)	0.049

Abbreviations: HR, hazard ratio; CI, confidence interval; TNBC, triple-negative breast cancer; PD, progressive disease; PR, partial response; SD, stable disease. Model adjusted for age, clinical stage, and histologic grade. Harrell’s C-index = 0.82.

## Data Availability

No new data were created or analyzed in this study.
